# Dr. N. S. Davis

**Published:** 1887-02

**Authors:** 


Dr. N. S. Davis completed his three-score years and ten on the 8th of January, and in honor of the event a large number of his friends assembled at his residence on that occasion to congratulate him, and to show their appreciation of the results of his long years of faithful and unselfish labor in the field of medicine.

Several addresses, appropriate to the occasion, were delivered, and he was the recipient of numerous handsome presents.

In replying to the addresses, Dr. Davis recalled the incidents of his early life; the struggles of the first years of his professional career, and the difficulties attending the organizing of the American Medical Association, of which he is conceded to be the father.

His triumphs over all obstacles teach a useful *lesson of life*, and show how much may be accomplished by one man where faithful and persistent effort is made to benefit his fellow man.

Beginning life amid obstacles that would have discouraged and deterred the average man from undertaking the task he assigned himself, he persevered until one by one they vanished before his tireless energy.

Having spent his earlier years in manual labor upon a farm, he worked his way into the medical profession, and, whilst advancing himself, he has done much to keep the medical profession of America advancing with him; and to-day, as President-Elect of the *Ninth International Medical Congress*, to be held in Washington in September of this year, he holds the highest post of honor in the medical profession of the world.

At home and abroad it is conceded that in conferring that honor on this veteran, it honored those who gave it as well as him who received it, and, best of all, who merited it; and the JOURNAL AND EXAMINER adds its congratulations to him who thus stands *primus inter pares*, and it reiterates the oft-expressed wish that he may long be spared to enjoy the just rewards of an honorable professional career.

